# Tollip promotes hepatocellular carcinoma progression via PI3K/AKT pathway

**DOI:** 10.1515/med-2022-0453

**Published:** 2022-04-01

**Authors:** Lu Huang, Qiong Yang, Huihong Chen, Zhenggeng Wang, Qi Liu, Shuhua Ai

**Affiliations:** Department of Gastroenterology, The Second Affiliated Hospital, Hengyang Medical School, University of South China, Hengyang City, 421001, Hunan province, China; Department of Gastroenterology, The Second Affiliated Hospital of University of South China, No. 35 Jiefang Road, Hengyang City, 421001, Hunan province, China

**Keywords:** Tollip, hepatocellular carcinoma, epithelial-mesenchymal transition, PI3K/AKT, prognosis

## Abstract

The activation of signaling pathways induced by Toll-like receptor (TLR) has been demonstrated to play essential roles in multiple liver diseases. Toll-interacting protein (Tollip) acts as an endogenous negative modulator of TLR signaling and is implicated in various cardio-metabolic diseases. However, the effect of Tollip in hepatocellular carcinoma (HCC) remains elusive. In the current study, enhanced Tollip expression was observed in HCC cells and tissues examined by RT-PCR, western blot, and immunohistochemistry staining. Moreover, the co-immunofluorescence staining demonstrated that increased Tollip expression was primarily located in hepatocytes. Functionally, Tollip overexpression significantly increased proliferation, migration, invasion, and epithelial-mesenchymal transition (EMT) of HCC cells, which ultimately accelerated tumorigenesis. Mechanistically, Tollip overexpression dramatically promoted the activation of PI3K/AKT signaling pathway in HCC cells which was attenuated by Tollip silencing. Importantly, the inhibition of PI3K/AKT axis can abolish the promoted effects of Tollip on proliferation and EMT of HCC cells. Our current study demonstrated that Tollip played an important role in the regulation of HCC development by engaging PI3K/AKT signaling pathway. These evidences suggested that the blockade of Tollip-PI3K/AKT axis was an ideal therapeutic treatment for management of HCC.

## 1 Introduction

Hepatocellular carcinoma is well recognized as one of the most common malignant disease in human with high rate of mortality and morbidity worldwide [1,2]. About more than 780,000 new liver cancer cases occur each year, more than 45–50% of the total number of cases and deaths are found in China, which is the second leading cause of cancer-related death [3,4]. Although great improvements have been made and widely applied in clinical treatment, including surgery, radiofrequency ablation, transcatheter arterial chemical embolism, and transplantation for local treatment, the actual survival improvement and prognosis of patients with HCC are still unfavorable because of the insidious onset, high invasion, rapid progression, and recurrence of metastasis [5,6,7,8]. Therefore, it is important to explore novel markers and unclose the underlying mechanism to improve HCC diagnosis and prognosis.

Toll-like receptor (TLR) family has been involved in multiple inflammatory-related cancers and differential expressions of TLRs are associated with poor outcome of HCC [9]. In the past decades, TLRs have emerged as playing crucial roles in HCC development and the underlying signaling pathway is responsible for mediating inflammation and HCC immunity responses [10,11]. The wide use of agonists and antagonists of TLRs renders it as the potential therapeutic target by activation of downstream molecules in regulation of initiation, progression, and metastasis of HCC [11,12]. Tollip is identified as an important endogenous modulator of TLR signaling and the structure within Tollip contains a Tom1 binding domain at the N-terminus, coupling ubiquitin to endoplasmic reticulum degradation domain at the C-terminus [13]. Recently, several studies have demonstrated that Tollip also participates in cardiovascular diseases. Overexpression of Tollip attenuates the hypertrophic response of neonatal cardiomyocytes through negative regulation of the MyD88-dependent NF-κB pathway [14]. Meanwhile, Tollip protects against chronic pressure overload-induced cardiac hypertrophy [15] and inhibits neointima formation by attenuating VSMC phenotypic switching, migration, and proliferation [16]. Neutralization of Tollip acts as a novel therapeutic target for hepatic ischemia-reperfusion injury in mice [17]. However, whether Tollip plays an essential role in HCC development and the underlying mechanism has not yet been investigated. Thus, we employed *gain-of-function* of Tollip in HCC cells to determine the specific role of Tollip in hepatocellular carcinoma.

In the current study, we observed that the Tollip exhibited a significant upregulated expression in hepatocellular carcinoma. The overexpression of Tollip exhibited oncogenic effect by promoting proliferation, migration, and metastasis of HCC cells. Mechanistically, Tollip overexpression activated PI3K/AKT signaling pathway and inhibition of PI3K/AKT significantly abolished the tumorigenic effect mediated by upregulated Tollip expression. Our data suggested that Tollip was a potential biomarker and therapeutic target for HCC management.

## 2 Materials and methods

### 2.1 Cell lines

The normal liver cell lines HL-7702 and Hepatic tumor cell lines, including Hep3B, Bel-7402, Huh7, and SMMC-7721 were purchased from the Cell Bank of Chinese Academy of Sciences (Shanghai, China). The cells were cultured in RPMI-1640, 10% fetal bovine serum (FBS) (Gibco), 1% penicillin, and streptomycin.

### 2.2 Animals and treatment

To generate HCC, 2 × 10^6^ HCC cells infected with Tollip overexpression were injected subcutaneously into 4-weeks-old BABL/c nude mice and the volume of the tumors was measured weekly (*n* = 6–8 each group). After 4 weeks, the mice received intraperitoneal anesthesia with pentobarbital sodium (50 mg/kg), and the tumors were collected and weighed. 1 × 10^6^ HCC cells infected with Tollip overexpression or control were implanted into nude mice via tail vein to establish the lung metastasis model (*n* = 6 each group). After 6 weeks, the lungs were collected and the number of metastatic lung nodules were calculated after staining with hematoxylin and eosin. All the procedures were performed according to the Guide for the Care and Use of Laboratory Animals published by the US National Institute of Health. The Animal Care and Use Committee of the Second Affiliated Hospital of University of South China approved all the study protocols.

### 2.3 RNA isolation and quantitative real-time PCR

Total RNA was extracted using a TRIzol reagent (Invitrogen) and cDNAs were synthesized using a Transcriptor First Strand cDNA Synthesis Kit (Roche, Indianapolis, IN). The data were identified by using quantitative real-time PCR with LightCycler 480 SYBR Green 1 Master Mix (Roche) and a LightCycler 480 QPCR System (Roche) in accordance with the manufacturer’s instructions. The results were shown after normalizing against GAPDH gene expression. The primer for Tollip detection is 5′-ATCTCCCCCATAAGAGTTTGAGTC-3′ and 5′-CACAGTTGGCATCAGGACCACAGGC-3′.

### 2.4 Western blotting analysis

The protein extracted from HCC Cells or tissues were lysed in ice-cold radioimmunoprecipitation assay buffer and then incubated with primary antibodies overnight at 4°C. The primary antibodies are as follows: Tollip (1:1,000, ab37155, Abcam), Total-AKT (1:1,000, 4691, Cell Signaling Technology), p-AKT (1:1,000, 4046, Cell Signaling Technology), Total-mTOR (1:1,000, BS1555, Bioworld Technology), p-mTOR (1:1,000, BS470, Bioworld Technology), E-Cadherin (1:1,000, ab231303, Abcam), N-Cadherin (1:1,000, ab76011, Abcam), Snail (1:1,000, ab180714, Abcam), Vimentin (1:1,000, ab92547, Abcam), and GAPDH (1:1,000, SC-25778, Santa Cruz). The relative expression was obtained using a FluorChem E Imager (ProteinSimple, FluorChem E), and membranes were treated with enhanced chemiluminescence reagents (170-5061; Bio-Rad). The results were shown after normalizing against GAPDH gene expression.

### 2.5 Immunofluorescence staining

The HCC tissues and cell sections were prepared according to the protocols. Following incubation with primary antibodies overnight at 4°C, the slides were washed with phosphate-buffered solution and incubated with the relevant secondary antibodies. The primary antibodies are as follows: Tollip (ab37155, Abcam) and HNF4 (ab41898, Abcam). The images were analyzed using Image-Pro Plus 6.0.

### 2.6 Establishment of Tollip overexpression in HCC cell lines

Human Tollip cDNAs were generated with the pBabe.puro retroviral constructs. The Tollip overexpression constructs were transfected into HCC cells by lipofectamine 2000 according to the protocol as previously described [18] and then selected by adding 400 µg/mL of G418 for 4 weeks to establish stable HCC cell lines.

### 2.7 Cell migration, invasion, and proliferation

Transwell chamber assay with or without Matrigel (BD)-coated transwell inserts were performed to assess the migration and invasion of HCC cells as described [18]. The upper chamber were plated with 2 × 10^5^ cells in 200 µL of serum-free medium, while 600 µL of medium with 10% FBS was added to the lower wells. After 24 h of incubation, the cells on the underside of the filter were fixed with 4% paraformaldehyde and stained with 0.1% crystal violet. The inserts were coated with 50 µL of 1 mg/mL Matrigel matrix according to the manufacturer’s recommendations to assess the invasive ability of HCC cells, which is similar to the migration assay. The capacity of HCC cells proliferation was analyzed using the cell counting kit-8 (CCK-8) according to the manufacturer’s protocol and the optical density was determined with microplate reader at a wave length of 450 nm.

### 2.8 Statistical analysis

Data were expressed as mean values ± SD. Comparisons between different groups were undertaken using the two-tailed Student *t* test or one-way ANOVA. All statistical analysis was performed with the SPSS 22.0 software and the statistical significance was *p* < 0.05.

## 3 Results

### 3.1 Enhanced Tollip expression in HCC

**Figure 1:**
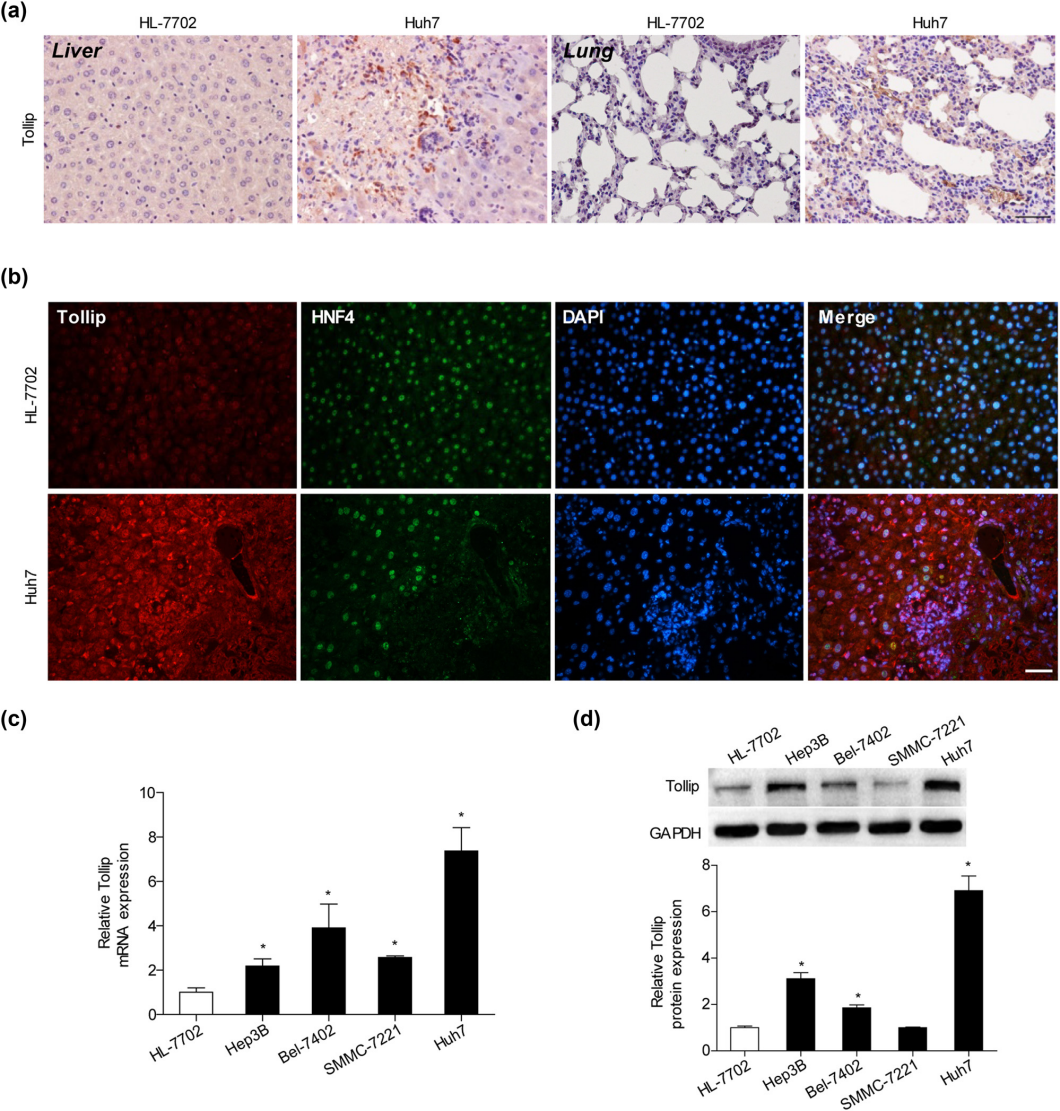
Upregulation of Tollip expression in HCC. (a) The expression of Tollip in HCC and lung tissues of BABL/c nude mice following subcutaneous injection of HL-7702 or Huh7 tested by immunochemistry staining. Scale bar = 50 µm. *n* = 5–10 fields per experimental group. (b) The immunofluorescence staining shows Tollip (red) expression and localized in hepatocyte identified by HNF4 (green) of liver tissues from BABL/c nude mice following subcutaneous injection of HL-7702 or Huh7. Scale bar = 50 µm. *n* = 6–8 fields per experimental group. (c and d) The expression of Tollip mRNA or protein level tested by RT-PCR or protein in HCC cell lines. *n* = 3 independent experiments. *p < 0.05 compared with control group.

To explore the potential role of Tollip in the development of HCC, the HCC cell lines and pathological tissues were analyzed to observe whether Tollip expression was changed. We observed that Tollip expression showed a stronger immunoreactivity in tissues of HCC and lung induced by Huh7 cells compared with HL-7702 controls (Figure 1a). The increased Tollip expression was primarily localized in hepatocyte identified by Hepatocyte nuclear factor 4 (HNF4) immunofluorescence staining (Figure 1b). Moreover, upregulated mRNA (Figure 1c) and protein (Figure 1d) expression of Tollip were found in HCC cell lines (Hep3B, Bel-7402, Huh7, and SMMC-7721) compared to normal liver cell line (HL-7702).

### 3.2 Tollip overexpression promotes tumorigenesis and proliferative capacity of HCC cells

**Figure 2:**
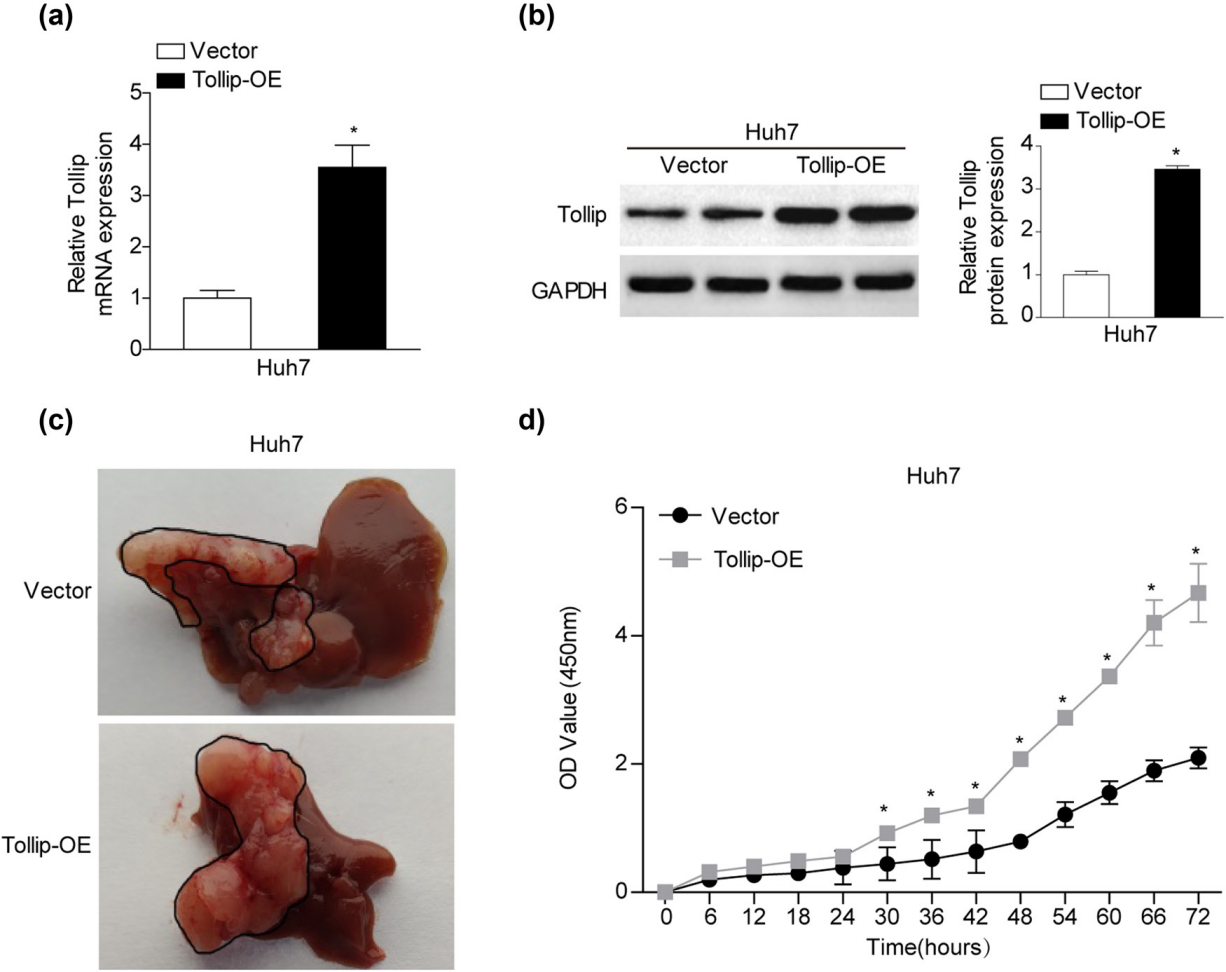
The oncogenic functions of Tollip in HCC. (a and b) The efficiencies of Tollip overexpression in HCC cell lines verified by RT-PCR (a) and western blot analysis (b). *n* = 3 independent experiments. (c) The morphology images of tumors following subcutaneous injection of Tollip-OE and vector. *n* = 5 mouse number. (d) Assessment of the influence of Tollip overexpression on HCC proliferation tested by CCK-8 at the indicated time. *n* = 3 independent experiments. *p < 0.05 compared with control group.

To determine whether the upregulated Tollip expression in HCC tissues and cells contributes to the development of HCC, the *gain-of-function* of Tollip on tumorigenic capacity of HCC *in vivo* and *in vitro* were further performed. Huh7 cells were stably transfected with Tollip overexpression vector. The mRNA and protein expression of Tollip in the above corresponding HCC cell lines were testified by RT-PCR and western blot (Figure 2a and b). First, we subcutaneously injected stable Huh7-Tollip-overexpression (Tollip-OE) and Vector into nude mice. The tumors in Tollip-OE group grew more rapidly compared with control group (Figure 2c). To testify our *in vivo* observation, we further investigated the biological function of Tollip in HCC progression *in vitro*. Compared with control groups, the CCK-8 analysis demonstrated that Tollip-OE exhibited significant increased ability of HCC cells proliferation (Figure 2d). These evidences indicate that Tollip is a potential important regulator in HCC tumorigenesis and growth.

### 3.3 Overexpression of Tollip promotes migratory, invasive, and metastatic capacities of HCC cells

**Figure 3:**
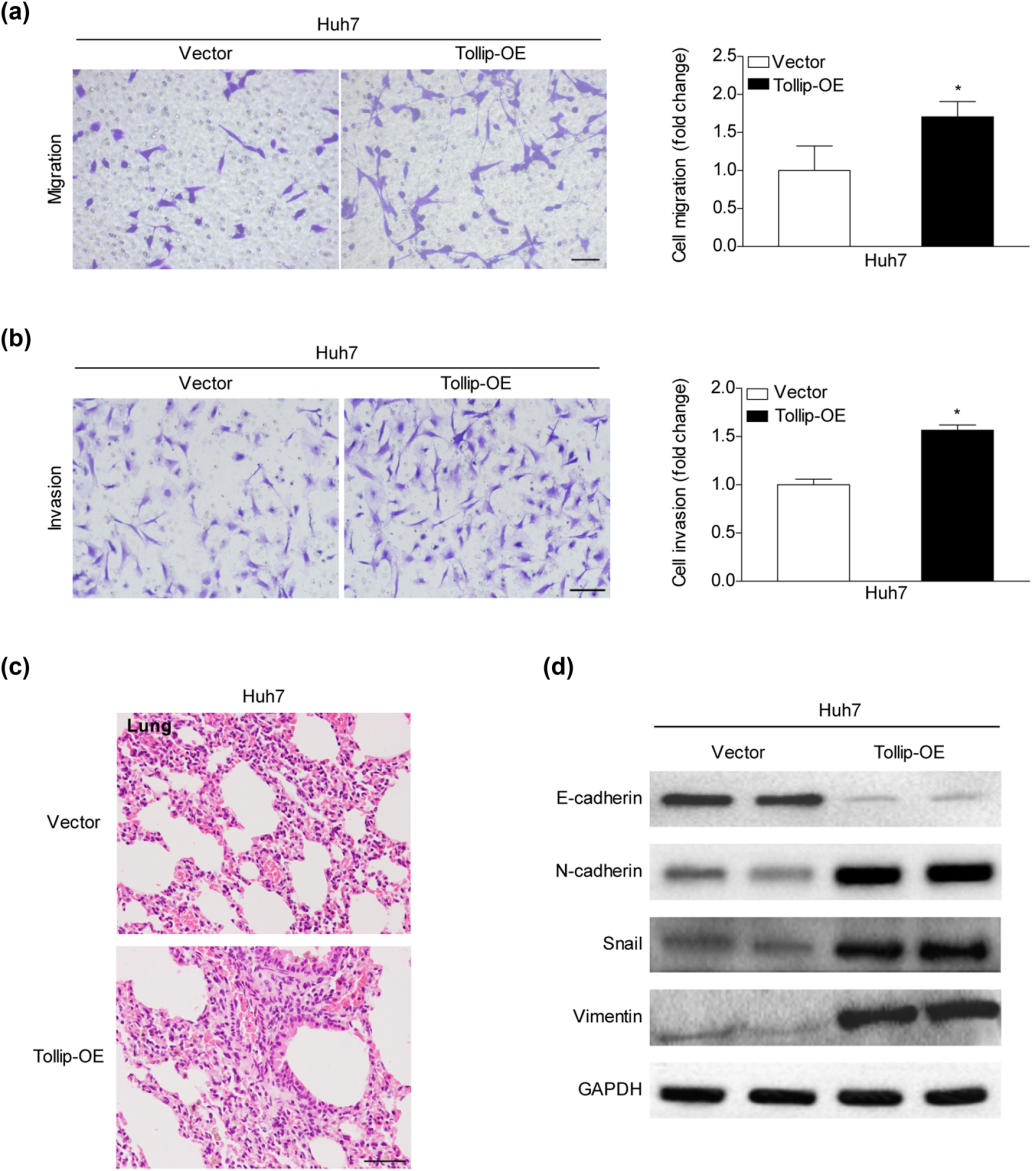
Tollip enhanced migratory, invasive, and metastatic capacities of HCC cells. (a) Evaluations of the influence of Tollip-OE on migration activities by transwell migration assays. *n* = 5 fields per experimental group. (b) Matrigel invasion assays for testing the effect of Tollip-OE on invasion ability. *n* = 5 fields per experimental group. (c) The lung slides from nude mice subjected with Tollip-OE detected by hematoxylin-eosin staining. Scale bar = 50 µm. *n* = 10 fields per experimental group. (d) Expressions of EMT-related markers in HCC cell lines infected with overexpression of Tollip by western blot assays. *n* = 3 independent experiments. *p < 0.05 compared with control group.

Next the Boyen’s chamber assay was performed to test the effect of Tollip on cell migration and invasion. Compared with the counterpart controls, the transwell assays showed that Tollip-OE remarkably promoted the migration ability of HCC cells (Figure 3a). As expected, we also demonstrated that the HCC cells transfected with Tollip-OE exhibited a more aggressive ability of invasion through Matrigel than the compared group (Figure 3b). Then, we injected Tollip-OE into nude mice via tail vein to investigate the metastatic potential by examination of tumor burden in the lung. The results showed that the metastatic foci in Tollip-OE mice dramatically enhanced in the lung sections (Figure 3c). The progression of epithelial-mesenchymal transition (EMT) is recognized as the key initiation step in metastasis [19]. We noticed that the increased Tollip expression significantly promoted the protein level of epithelial marker expression, whereas attenuated the mesenchymal marker expression in the HCC cells infected with Huh7 (Figure 3d). Together with the above results, these data suggested the critical pro-tumorigenic role of Tollip in HCC.

### 3.4 Tollip promotes PI3K/AKT signaling pathway in HCC cells

**Figure 4:**
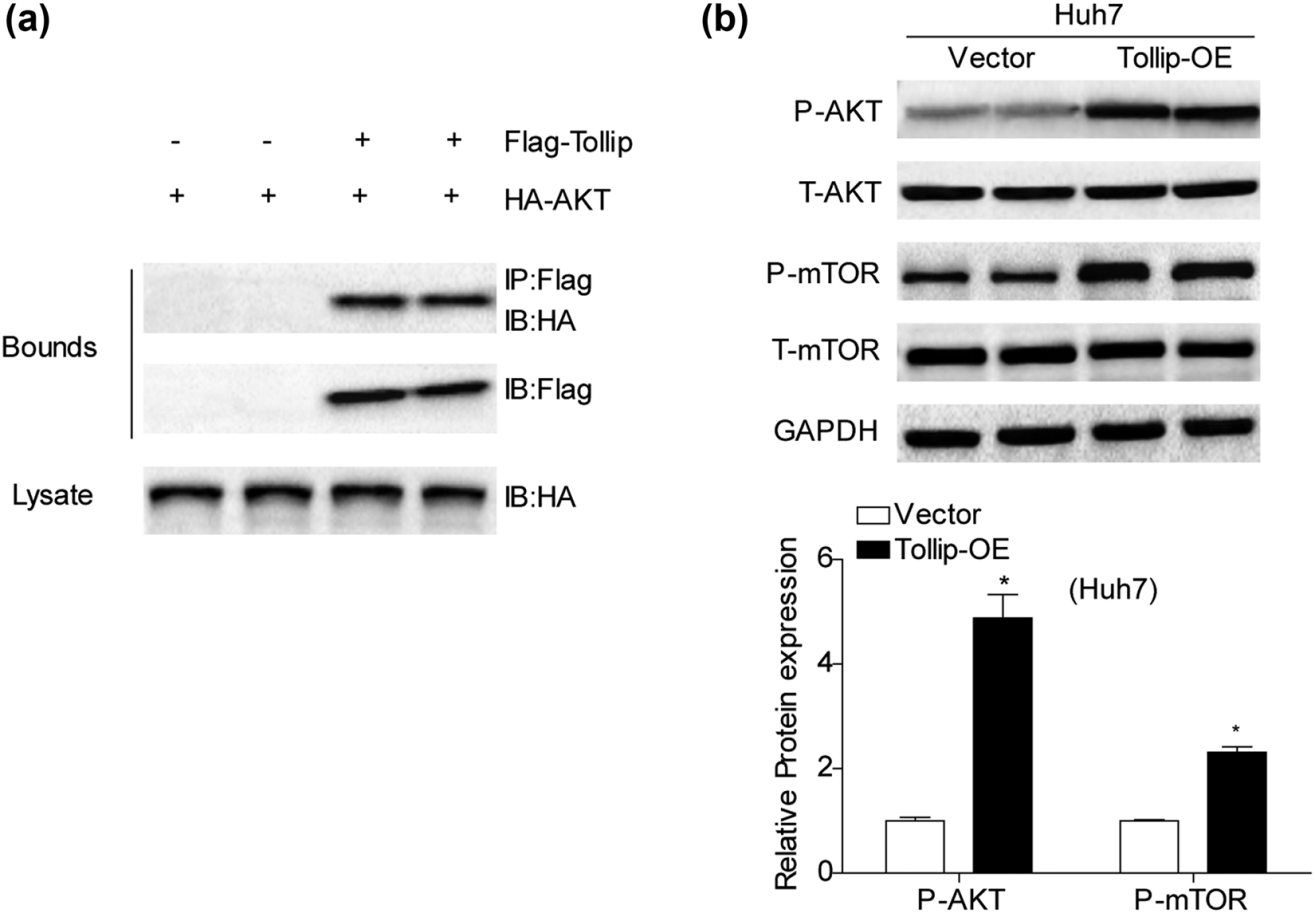
The regulation of PI3K/AKT signaling pathway in HCC cells by Tollip. (a) Western blots were performed with Tollip or AKT antibody after co-IP of Tollip. (b) Immunoblot showed AKT and mTOR phosphorylation and total levels in Tollip-OE and vector group. *n* = 3 independent experiments. *p < 0.05 compared with control group.

To identify the underlying mechanism by which Tollip promoted HCC development, we next investigated the activation of PI3K/AKT/mTOR signaling pathway in HCC, which was well recognized as important role in tumorigenesis [20]. The co-immunoprecipitation experiments were performed and the results showed that Tollip could interact with AKT (Figure 4a). Moreover, we observed that the activation of PI3K/AKT pathway exhibited a stronger immunoblot activity in HCC cells transfected with Tollip-OE, which were characterized by a significant increase in AKT and mTOR phosphorylation expression, respectively (Figure 4b). These data suggested that Tollip may exert its pro-tumorigenesis effects by activation of PI3K/AKT signaling pathway.

### 3.5 PI3K/AKT signaling is required for the function of Tollip in HCC cells

**Figure 5:**
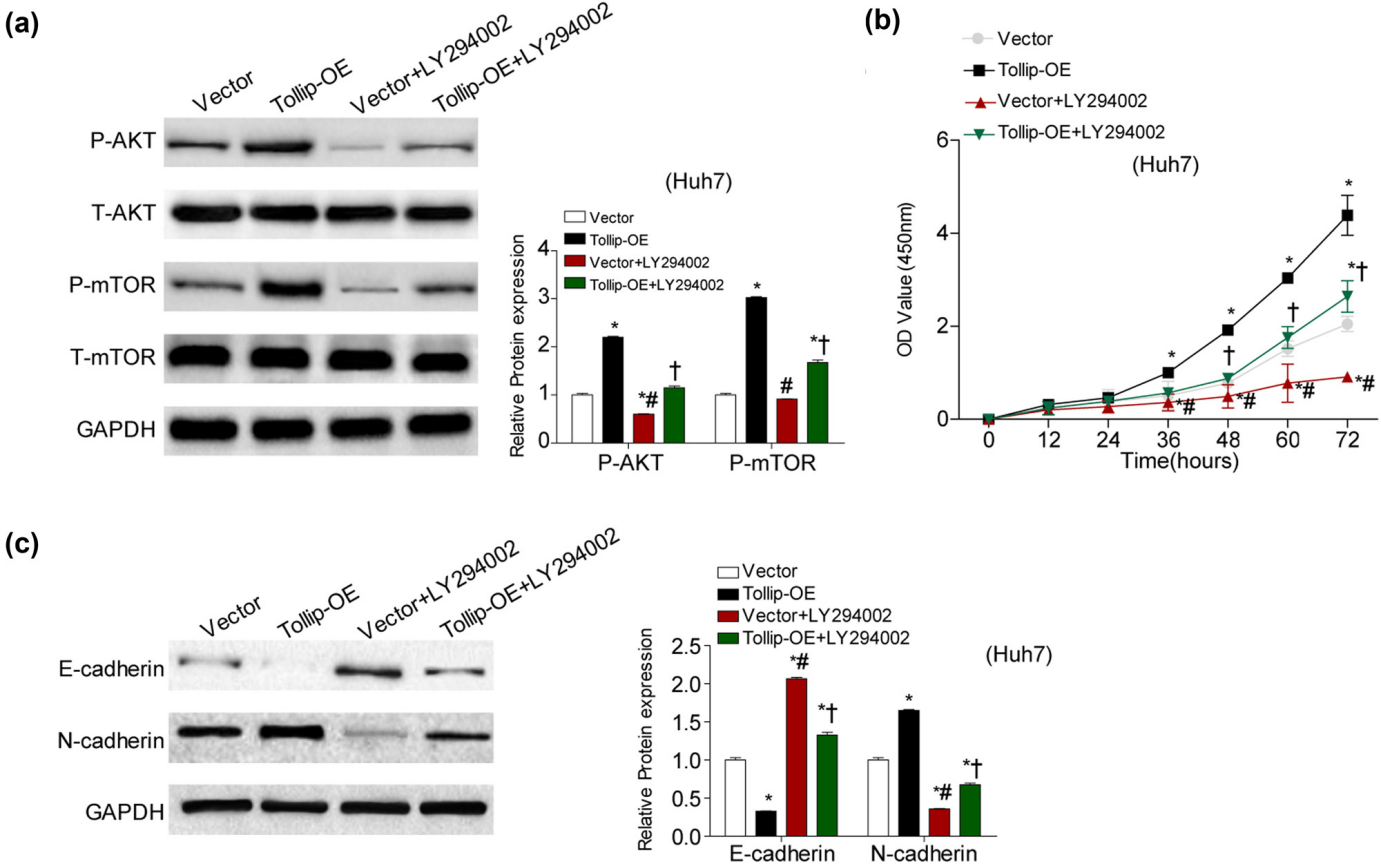
PI3K/AKT activation is required for the pro-tumorigenic properties of Tollip. (a) Immunoblot showed AKT and mTOR phosphorylation and total levels in HCC cells infected with Tollip-OE pretreated with LY294002. *n* = 3 independent experiments. (b and c) The impact of inactivation of PI3K/AKT pathway by LY294002 in Tollip-OE mediated cell proliferation (b) and EMT (c). *n* = 3 independent experiments. *p < 0.05 compared with Vector group. #*p* < 0.05 compared with Tollip-OE group. †*p* < 0.05 compared with Vector with LY294002 group.

Next to determine whether the effect of Tollip on HCC development was dependent on PI3K/AKT signaling activation, the HCC cells transfected with Tollip-OE were treated with LY294002 (an inhibitor for PI3K/AKT pathway), which had been testified by western blot (Figure 5a). We demonstrated that the effects of tumorigenesis mediated by Tollip-OE were largely abolished by negative activation of AKT, as evidenced by decreased ability of proliferation (Figure 5b) and EMT of HCC cells (Figure 5c). Taken together, the results indicated the regulation of tumorigenesis by Tollip may be partially mediated by activation of AKT signaling pathway.

## 4 Discussion

By using a variety of *gain-of-function* of Tollip *in vivo* and *in vitro* approaches, our current study demonstrated that Tollip was an independent prognostic indicator for HCC management, which showed an upregulation of Tollip in HCC tissues and exhibited the clinical significance and functional regulation in tumorigenesis. Tollip overexpression in HCC cells induced tumor formation and growth, characterized by enhanced proliferation, migration, invasion, and metastasis of HCC cells, as well as positively regulated the transition between epithelial and mesenchymal capacities in HCC cells. Importantly, we demonstrated that Tollip promoted PI3K/AKT signaling pathway in HCC cells and the pro-tumorigenic effects of Tollip were largely abolished by negative activation of PI3K/AKT signaling. Thus, we proposed a novel model for Tollip activation of proliferative, migrative, invasive, metastatic, and EMT in HCC cells, at least partially through activation of PI3K/AKT signaling.

TLRs are well published to be expressed by a wide variety of cell types including immune cells, macrophage, and cardiovascular cells [15,16,21,22]. Previous studies have demonstrated that TLRs play crucial roles in regulation of biological processes such as innate immune responses, the induction of adaptive immune responses, regulation of inflammation, would healing, and carcinogenesis [9,11,22]. In the past decades, TLR family members have emerged to involve in multiple inflammatory-related cancers. Specifically, differential expressions of TLRs are found and function differently on different tumor cells. The association between TLR3, TLR4, and TLR9 expressions and tumor aggressiveness and poor prognosis in HCC was obtained from 30 patients with HCC [23]. Downregulation of TLR7 expression is observed in hepatitis-virus-related human HCC and TLR2 single-nucleotide polymorphism is required for HCC susceptibility [24]. More importantly, the changes in TLRs’ activities exert pro- or anti-tumor effect in HCC cells, while the underlying signaling pathway of TLRs is responsible for mediating inflammation and HCC immunity responses [9,10,11,25]. Thus, the application of agonists and antagonists of TLRs provide novel promising therapeutic target for HCC therapy. TLRs can be stimulated by multiple invading exogenous pathogens through PAMPs and recognized endogenous ligands released by necrotic cells through damage-associated molecular patterns (DAMPs), containing high-motility group box-1, heat shock protein, fibrinogen, heparin sulfate, fibronectin, hyaluronic acid, and double- and single-strand RNA, that have been demonstrated to promote tumor cell survival and proliferation by targeting various TLRs expressed on tumor cells and subsequently activate the key downstreams signaling pathways of TLRs to affect the development of HCC [26]. Although Tollip may act as potential regulator for mediating the effect of interaction of TLRs and their ligands on tumorigenesis, there are limited evidences about the direct relationship between Tollip and such ligands. Accumulative evidences have demonstrated that TLRs’ stimulation leads to the activation of multiple signaling pathways, including NF-κB, MAPK, Jun N-terminal kinases, P38, and extracellular signal-regulated kinase, as well as several interferon regulatory factors, which result in activation of inflammatory cytokines implicated in hepatocellular carcinoma. Additionally, TLR activation in antigen-presenting cells can also trigger adaptive immunity to participate in hepatocellular carcinoma [27]. Tollip acts as an ubiquitin-binding protein which can interact with several components of the TLR signaling implicated in immune response [13,15]. Besides, recent studies have shown that Tollip has a negative effect on TLR signaling and plays an important role in cardio- and metabolic-diseases. Tollip deficiency accelerates vascular smooth muscle cell-mediated intimal hyperplasia by activation of AKT signaling [16] and promotes ventricular hypertrophy induced by pressure overload [15]. Meanwhile, Tollip also acts as a key regulator of hepatic IR injury by interaction with ASK1 which leads to activation of c-Jun N-terminal kinase/p38 signaling [17]. Due to its altering regulatory function under certain pathophysiological conditions and the important regulation of key downstreams of TLRs, we thus examined whether Tollip can exert crucial role during HCC. Previous studies exhibit that Tollip shows significant ubiquitination change in Hepatitis B virus (HBV)-integrated HepG2.2.15 model cell line compared with HepG2 group such that HBV infection remains the leading cause of HCC, while the expression of Tollip is closely correlated with the hepatic Ischemia-Reperfusion process and Tollip deficiency increases liver steatosis [17,28,29]. Importantly, among a variety of tumor cells tested in our current study, we found that the expression of Tollip showed the greatest difference and the significantly increased trend in Huh7 cells. The results indicated that the dramatically upregulated expression of Tollip in Huh7 cells exhibited the tight correlation with the occurrence and development of HCC, suggesting that Huh7 cells were the best cell model for studying the effect of Tollip on regulation of the occurrence and development of HCC. Previous studies demonstrated that Tollip deficiency enhanced tumor immune surveillance through neutrophil reprogramming. However, our current study showed that the overexpression of Tollip exhibited oncogenic effect by promoting proliferation, migration, and metastasis of HCC cells by activation of PI3K/AKT signaling pathway. These evidences provide important evidences of flexibility, versatility, and potency of Tollip in regulating different diseases and cell lines upon different pathological stimulus by engaging the individual downstream targets, and exerts even opposite effect.

Abnormal AKT activation is a hallmark of tumor progression in various cancers, including HCC. Among them, the PI3K/AKT signaling pathway is one of the most frequently altered signaling networks in human cancers because of the critical regulation of cell proliferation, survival, growth, and motility, and has become an attractive target in anticancer therapy [20,30,31]. Previous studies have identified cross talks between differential TLR signaling and the PI3K/Akt pathway in HCC development [20,32,33]. To investigate the molecular mechanism by which Tollip overexpression accelerated the development of HCC, we therefore examined the status of the PI3K-AKT signaling pathway. An important finding of this study was that PI3K-AKT activation was promoted by Tollip overexpression. Several drugs targeting PI3K/AKT pathway in HCC are currently in different phases of clinical trials. The most direct approach to inhibit PI3K/AKT pathway is to directly target PI3K itself and such inhibitors, including LY294002 and wortmannin targeting the catalytic site of p110, have been extensively used as effective research tools for investigation of PI3K/AKT in HCC [34,35]. By using LY294002, we noticed that the pro-tumorigenic effects of Tollip overexpression were largely dependent on the activation of PI3K-AKT signaling. Together, these results indicated that the activation of PI3K-AKT axis was a major mechanism underlying the Tollip-elicited tumorigenic effects in HCC.

Our study demonstrated that Tollip acted as critical driver of HCC development for the first time. Functional experiments established the essential role of Tollip in promoting HCC aggression and metastasis, identified by increased proliferation, migration, invasion, and EMT through PI3K-AKT signaling. Furthermore, inhibition of PI3K-AKT signaling significantly attenuated the effect of Tollip on tumorigenesis. These findings demonstrated the importance of Tollip in HCC and revealed that targeting of Tollip may be a promising novel therapeutic strategy for HCC prognosis and treatment.

## Figures and Tables

**Figure A1:**
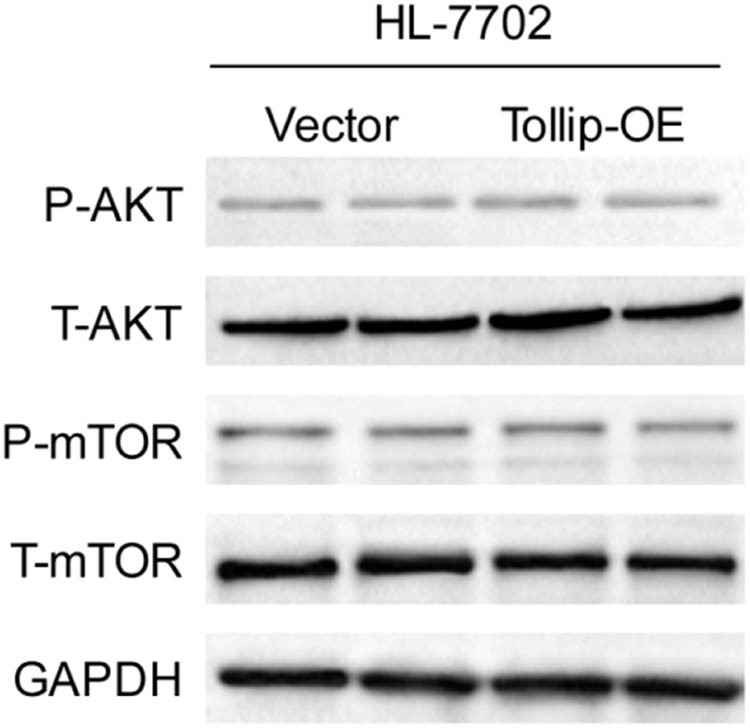
The regulation of PI3K/AKT signaling pathway in HL-702 cells by Tollip. Immunoblot showed AKT and mTOR phosphorylation and total levels in HL-7702 infected with Tollip-OE and vector group. *n* = 3 independent experiments. *p < 0.05 compared with control group.

**Figure A2:**
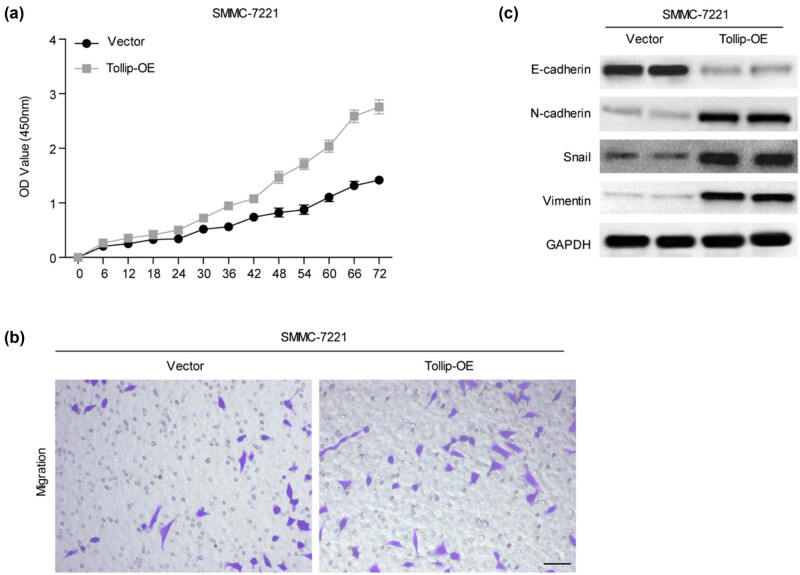
Tollip enhanced proliferate, migratory and metastatic capacities of SMMC-7221 cells. (a) Assessment of the influence of Tollip overexpression on SMMC-7221 proliferation tested by CCK-8 at the indicated time. (b) Evaluations of the influence of Tollip-OE on migration activities of SMMC-7221 by transwell migration assays. *n* = 5 fields per experimental group. (c) Expressions of EMT-related markers in SMMC-7221 cell infected with overexpression of Tollip by western blot assays. *n* = 3 independent experiments. *p < 0.05 compared with control group.

**Figure A3:**
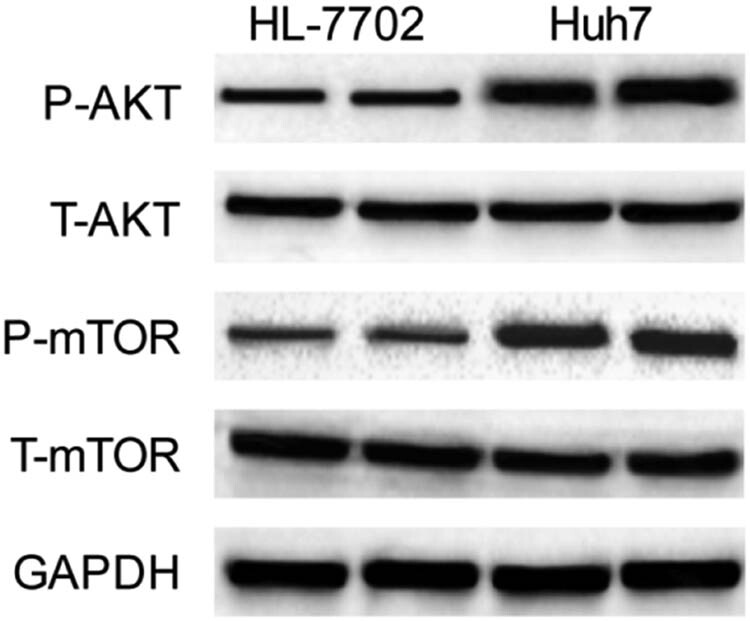
The activation of PI3K/AKT signaling pathway in HL-7702 and Huh7 cells. Immunoblot showed AKT and mTOR phosphorylation and total levels in HL-7702 and Huh7 group. *n* = 3 independent experiments. *p < 0.05 compared with control group.
