# Erratum to “Tollip promotes hepatocellular carcinoma progression via PI3K/AKT pathway”

**DOI:** 10.1515/med-2022-0501

**Published:** 2022-06-11

**Authors:** Lu Huang, Qiong Yang, Huihong Chen, Zhenggeng Wang, Qi Liu, Shuhua Ai

**Affiliations:** Department of Gastroenterology, The Second Affiliated Hospital, Hengyang Medical School, University of South China, No. 35 Jiefang Road, Hengyang City, 421001, Hunan Province, Hunan, China

In the published article “Huang L, Yang Q, Chen H, Wang Z, Liu Q, Ai S. Tollip promotes hepatocellular carcinoma progression via PI3K/AKT pathway. Open Med (Wars). 2022 Apr 1;17:626–37. doi: 10.1515/med-2022-0453. PMID: 35434373; PMCID: PMC8976180” the funding information is incomplete, and the affiliation of the authors in incorrect.

The correct affiliation of authors is as follows:

Lu Huang, Qiong Yang, Huihong Chen, Zhenggeng Wang, Qi Liu, Shuhua Ai*:

Department of Gastroenterology, The Second Affiliated Hospital, Hengyang Medical School, University of South China, Hengyang, Hunan, 421001, Hunan, China

*Corresponding author: Shuhua Ai, Department of Gastroenterology, The Second Affiliated Hospital, Hengyang Medical School, University of South China, No. 35 Jiefang Road, Hengyang City, Hunan Province, 421001, Hunan, China, e-mail: shuhua_ai_usc@163.com

Full funding information is as follows:

This work was supported by grants from the Human Health and Family Planning Commission Research Fund of Hunan Province (no. A2017014), Guiding scientific research project of Hengyang Science and Technology Bureau (2019-174), and Scientific Research Project of Health Commission of Hunan Province, China (202203034243).

